# Left Atrial Appendage Morphology Predicts Atrial Fibrillation Recurrence: The Hidden Risks of Windsock Anatomy

**DOI:** 10.3390/diagnostics15202642

**Published:** 2025-10-20

**Authors:** Yu-Sheng Lin, Hui-Ting Wang, Yen-Nan Fang, Huang-Chung Chen, Yi-Wei Lee, Yung-Lung Chen

**Affiliations:** 1 Section of Cardiology, Department of Internal Medicine, Kaohsiung Chang Gung Memorial Hospital, Kaohsiung 833401, Taiwan; samm3y5@cgmh.org.tw (Y.-S.L.); wideopen@cgmh.org.tw (Y.-N.F.); chc3@cgmh.org.tw (H.-C.C.); 2 Emergency Department, Kaohsiung Chang Gung Memorial Hospital, Kaohsiung 833401, Taiwan; gardinea1983@gmail.com; 3 School of Medicine, College of Medicine, National Sun Yat-Sen University, Kaohsiung 804201, Taiwan; 4 Department of Radiology, Kaohsiung Chang Gung Memorial Hospital, Kaohsiung 833401, Taiwan; 5 Graduate Institute of Clinical Medical Sciences, College of Medicine, Chang Gung University, Taoyuan 333323, Taiwan

**Keywords:** atrial fibrillation recurrence, cardiac computed tomography, catheter ablation, left atrial appendage morphology, windsock anatomy

## Abstract

**Background/Objectives**: Left atrial appendage (LAA) morphology has been implicated in atrial fibrillation (AF) recurrence following catheter ablation. However, the predictive value of specific anatomical shapes remains unclear. We aimed to evaluate the association between distinct LAA morphologies and AF recurrence post-ablation. **Methods**: In this retrospective, single-center study, 463 patients with AF undergoing first-time catheter ablation were included. Pre-ablation contrast-enhanced cardiac computed tomography was performed to classify LAA morphology into chicken-wing, windsock, cauliflower, and cactus types. Patients were followed for one year, with AF recurrence defined as documented atrial tachyarrhythmia episodes lasting more than 30 s occurring between 3 and 12 months post-procedure. Clinical, anatomical, and procedural factors were analyzed using multivariable logistic regression to identify independent predictors of recurrence. **Results**: Among the four morphologies, the windsock-type LAA had the highest recurrence rate at 48.3%, significantly greater than chicken-wing (25.2%), cauliflower (20.8%), and cactus (18.2%) types (*p* = 0.017). Multivariable analysis confirmed windsock morphology as an independent predictor for AF recurrence (adjusted OR = 2.720, 95% CI: 1.209–6.118; *p* = 0.016). Additionally, persistent AF (adjusted OR = 1.748, 95% CI: 1.075–2.842; *p* = 0.024) and antiarrhythmic drug use in the blanking period (adjusted OR = 2.862, 95% CI: 1.689–4.849; *p* < 0.001) independently increased the risk of recurrence. **Conclusions**: Windsock-type LAA morphology significantly predicts increased AF recurrence following catheter ablation, underscoring the importance of morphological assessment in ablation planning. Individualized strategies targeting high-risk LAA morphologies may enhance procedural success and reduce AF recurrence. Future prospective studies are warranted to validate these findings.

## 1. Introduction

In addition to pulmonary vein (PV) triggers, the left atrial appendage (LAA) has been identified as an important source of ectopic activity contributing to atrial fibrillation (AF) recurrence. Previous studies have shown that approximately 27% of AF triggers arise from the LAA and in about 8% of patients. The LAA may even be the sole source of AF initiation [1]. Motivated by such findings, some ablation strategies have targeted the LAA itself: in one trial of patients with long-standing persistent AF, empirical electrical isolation of the LAA significantly improved freedom from AF during follow-up [2]. However, routine LAA isolation remains controversial [3]. Not all studies have demonstrated a clear benefit, and there is concern that rendering the appendage electrically silent without occlusion could promote stagnant flow and thrombus formation [4,5]. For instance, patients with incidental LAA isolation during ablation have been reported to experience a higher rate of thromboembolic events (about 17% over 4 years) compared to those without LAA isolation (4%), despite guideline-directed anticoagulation [6]. These mixed results underscore the need to better understand the conditions under which the LAA contributes to AF maintenance and recurrence.

The arrhythmogenic propensity of the LAA may be attributable to its unique anatomical and electrophysiological characteristics. The LAA is lined with abundant pectinate muscles that create a heterogeneous myocardial fiber orientation, which can alter local conduction and facilitate ectopic impulse formation [7]. Additionally, the LAA lies in close proximity to the ligament of Marshall and the Bachmann bundle—structures containing autonomic innervation and conductive tissue—and abnormal connections involving these regions can further contribute to ectopic firing from the LAA [8]. Furthermore, the persistence of AF itself leads to progressive atrial remodeling. AF is associated with left atrial dilation and wall stress changes, and over time, persistent AF promotes interstitial fibrosis and other structural alterations, which in turn increase the likelihood of AF recurrence after ablation. These observations suggest that both trigger (focal ectopy from the LAA) and substrate (atrial remodeling) factors related to the LAA play a role in arrhythmia recurrence.

Multiple studies have explored specific LAA features as predictors of post-ablation outcomes. For example, a larger LAA ostium (orifice) area has been associated with a higher risk of AF recurrence after catheter ablation [9]. Similarly, in patients with persistent AF, a greater LAA volume has been shown to predict AF recurrence regardless of whether radiofrequency or cryoballoon ablation is used [10]. An enlarged LAA often reflects more extensive atrial remodeling, and consistent with this, a low LAA flow velocity has also been correlated with arrhythmia recurrence [9]; notably, reduced appendage emptying velocity is a known risk factor for thromboembolism in AF patients. These findings highlight that the size and function of the appendage can influence ablation outcomes.

Despite the evidence linking LAA size and function to AF recurrence, the role of LAA morphology—the anatomical shape of the appendage—remains insufficiently explored. Prior investigations into LAA-based interventions generally did not stratify patients by appendage morphology, leaving it unclear whether certain LAA shapes are more arrhythmogenic or prone to recurrence than others. To date, the few studies that have examined LAA morphology in this context were limited by relatively small sample sizes. For example, one single-center study involving 84 patients suggested that chicken-wing LAA morphology carried the highest risk of post-ablation AF recurrence, with a recurrence rate of approximately 68% and an odds ratio of nearly 8 compared to other morphologies [11]. We hypothesized that distinct LAA morphologies (e.g., chicken-wing, windsock, cauliflower, cactus) might differentially impact the propensity for AF to recur after ablation. Therefore, this study aimed to investigate whether LAA morphology is associated with AF recurrence following catheter ablation.

## 2. Materials and Methods

### 2.1. Study Design and Population

This retrospective, single-center study was conducted at Kaohsiung Chang Gung Memorial Hospital to evaluate the association between LAA morphology and AF recurrence following catheter ablation. Eligible participants were adult patients aged 18 years or older with a confirmed diagnosis of AF who were undergoing their first ablation procedure. Patients were excluded if they lacked preprocedural contrast-enhanced cardiac computed tomography (CT), if the LAA could not be identified on imaging, or if they had less than 12 months of clinical follow-up after the ablation. Additional exclusion criteria included pregnancy, a prior history of surgical repair of atrial septal defect, the presence of LAA thrombus, or known hypersensitivity to iodinated contrast agents. All patients provided informed consent for the catheter ablation procedure, and the institutional review board of Chang Gung Medical Foundation approved the study protocol, granting a waiver of additional consent for retrospective data analysis (IRB number: 202500634B0, date: 28 April 2025).

### 2.2. LAA Morphology and Imaging Analysis

All patients underwent contrast-enhanced, ECG-gated cardiac CT scanning prior to ablation to delineate left atrial and LAA anatomy. Cardiac multislice detector computed tomography (MDCT) was performed using a 256-slice scanner (Somatom Definition Flash, Siemens Healthineers, Erlangen, Germany). With patients in the supine position, cardiac MDCT was conducted in the craniocaudal direction during a single breath-hold at end-inspiration. Scanning parameters, adjusted according to patient body size, were as follows: collimation, 256 × 0.6 mm; gantry rotation time, 0.28 s; pitch, 0.2–0.5; medium soft-tissue convolution kernel (B36); tube voltage, 120 kV; and tube current, 350–500 mA. Images were reconstructed with retrospective ECG gating at the end-systolic phase.

The scanning delay was determined using an automated bolus-tracking technique. An initial unenhanced scan was obtained at the level of the ascending aorta, 20 mm above the coronary ostia, to place a circular region of interest (ROI, 10 mm in diameter) within the aortic lumen. Based on patient body weight, 70–90 mL of nonionic contrast medium (Omnipaque 350, 350 mg I/mL; GE Healthcare, Cork, Ireland) was injected at a rate of 4–5 mL/s, followed by a 30 mL saline chaser at the same rate. Image acquisition was initiated automatically when the ROI attenuation exceeded 90 Hounsfield units (HU).

Reconstructed images had a slice thickness of 0.75 mm, an increment of 0.4 mm, and a matrix size of 512 × 512. The field of view (FOV) was manually adjusted to include the entire heart and pulmonary veins. All image data were transferred to a dedicated workstation equipped with post-processing software (Vitrea 7.14, Vital Images Inc., Minnetonka, MN, USA) for subsequent analysis.

**Figure 1:**
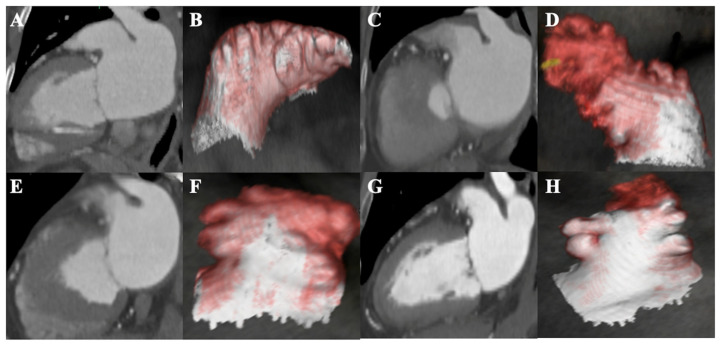
Left atrial appendage (LAA) morphology based on cardiac computed tomography (CT). Representative images of LAA morphologies from contrast-enhanced cardiac CT: axial views (**A**,**C**,**E**,**G**) and 3D volume-rendered reconstructions (**B**,**D**,**F**,**H**). (**A**,**B**) Chicken wing: Dominant lobe with a sharp bend near the proximal or mid segment. (**C**,**D**) Windsock: Single elongated dominant lobe with smooth curvature, lacking a sharp bend. (**E**,**F**) Cauliflower: Short, broad-based structure with no dominant lobe and multiple complex lobes near the ostium. (**G**,**H**) Cactus: Central dominant lobe with multiple secondary lobes projecting in superior and inferior directions, forming a forked appearance.

LAA morphology was classified into four conventional categories—chicken-wing, windsock, cauliflower, and cactus—based on established definitions (Figure 1). A chicken-wing LAA features a prominent dominant lobe with a sharp bend or fold in its proximal or mid portion. A windsock LAA has one dominant, elongated lobe (often with secondary or tertiary lobes) extending generally in an inferior direction. A cauliflower LAA is characterized by a short, complex structure with no single dominant lobe and multiple convoluted lobes near the ostium (often with an irregular or oval orifice). The cactus LAA consists of a dominant central lobe with multiple secondary lobes projecting both superiorly and inferiorly, giving it a forked, multibranched appearance [12,13]. Two independent reviewers (one cardiac radiologist and one cardiac electrophysiologist), who were blinded to the patients’ clinical outcomes, classified each patient’s LAA morphology; any disagreements were resolved by joint consensus review. In addition to morphological type, quantitative LAA anatomical parameters were measured from the CT images. These included: (1) the LAA orifice size, defined as the maximum short-axis diameter across the LAA ostial plane; (2) LAA length, defined as the longitudinal distance from the ostial plane to the tip of the dominant lobe; (3) the number of LAA lobes, counting any secondary lobe protruding >10 mm from the main lobe; (4) the distance from the LAA ostial midpoint to the left superior PV (LSPV) ostial midpoint; and (5) the LAA volume [12]. LAA volume was measured by manual planimetry of the LAA chamber on sequential axial CT slices, with the total volume calculated using the Simpson’s method on a dedicated 3D workstation. Left atrial volume (excluding the LAA) was similarly measured from the same CT dataset.

### 2.3. Catheter Ablation Procedure

All ablation procedures were performed under conscious sedation and local anesthesia. Vascular access was obtained via the femoral vein, and transseptal puncture was guided by intracardiac echocardiography or fluoroscopy to access the left atrium. A three-dimensional electroanatomic map of the left atrium was constructed using the EnSite Precision mapping system (Abbott, Chicago, IL, USA). Wide-area circumferential ablation lesions were delivered around the left and right PV antra to achieve PV isolation (PVI) as the initial lesion set. If the patient remained in AF after PVI, electrical cardioversion was performed to restore sinus rhythm. Isoproterenol infusion (up to a target dose as per standard protocol) was then administered to provoke any non-PV triggers; any induced focal atrial tachycardia (AT) triggers or ectopic firing sites (including those originating from the LAA or other atrial regions) were mapped and ablated. If any organized AT or atrial flutter (AFL) was observed during the procedure, activation mapping and targeted ablation were performed (including cavotricuspid isthmus ablation for cavotricuspid isthmus-dependent AFL if it was clinically documented or induced). In patients with persistent AF or those who had recurrent AF immediately after cardioversion without identifiable triggers, additional substrate modification was undertaken. This stepwise approach included empirical left atrial posterior wall isolation, ablation of complex fractionated atrial electrograms, and ethanol infusion in the vein of Marshall, applied sequentially until AF could no longer be induced or sustained.

### 2.4. Follow-Up and Outcome Measures

After ablation, patients were followed closely to assess rhythm outcomes. Follow-up visits were scheduled at approximately 1 week post-discharge, and at 1, 3, 6, 9, and 12 months after the procedure. At each visit, patients underwent a physical examination and a 12-lead electrocardiogram (ECG). Additionally, 24-h Holter monitoring was performed if patients reported symptoms suggestive of arrhythmia (e.g., palpitations) or at the 3- or 6-month visits in those without symptoms, per the physician’s discretion. The first 3 months following the ablation were defined as the blanking period, during which any recurrence of atrial arrhythmia was not considered a failure of the procedure. Antiarrhythmic drugs (AAD) (including amiodarone, propafenone, dronedarone, or flecainide) were permitted during the blanking period to manage symptomatic transient recurrences. The primary outcome was one-year AF recurrence post-ablation, excluding the blanking period, defined as any atrial tachyarrhythmia (AF, AFL, or AT) lasting >30 s. Any arrhythmia episode occurring during the blanking period was recorded for analysis, but these recurrences were not counted as endpoint events.

### 2.5. Statistical Analysis

Data were summarized and analyzed to determine associations between LAA morphology (and other variables) and 12 months post-ablation AF recurrence. Continuous variables are presented as mean ± standard deviation and were compared across LAA morphology groups using one-way analysis of variance (ANOVA). Categorical variables are expressed as counts and percentages and were compared using chi-square tests (or Fisher’s exact test, if appropriate). Freedom from atrial arrhythmia recurrence over time was estimated using the Kaplan–Meier method, and survival distributions were compared between groups with the log-rank test. To identify independent predictors of AF recurrence at 1 year, univariable logistic regression was first performed for all collected clinical, imaging, and procedural variables. Variance inflation factors (VIFs) were assessed to evaluate the presence of multicollinearity among the independent variables included in the regression models. Variables showing a *p*-value < 0.1 in univariable analysis were then entered into a multivariable logistic regression model (using a backward stepwise method) to determine which factors remained significant independent predictors. Results of the logistic regression are reported as odds ratios (ORs) with 95% confidence intervals (CIs). Nagelkerke’s R^2^ statistic was examined to evaluate the model’s explanatory power and overall predictive performance. A multivariate Cox proportional hazards regression model was employed to assess the independent associations between covariates and the outcome of interest. Results were expressed as hazard ratios (HRs) with corresponding 95% CIs. For all analyses, a two-sided *p*-value < 0.05 was considered statistically significant. Statistical analyses were carried out using SPSS version 25.0 (IBM Corp., Armonk, NY, USA).

## 3. Results

### 3.1. Patient Characteristics

Between 15 January 2013 and 6 September 2023, a total of 512 patients with AF were screened. After excluding 48 patients lost to follow-up and 1 patient without a measurable LAA on imaging, 463 patients were included in the study. The mean age of the cohort was approximately 61 ± 11 years, and 62% were male. Based on cardiac CT LAA morphology, patients were categorized into four groups: chicken-wing (*n* = 326), windsock (*n* = 29), cauliflower (*n* = 53), and cactus (*n* = 55). Interobserver concordance in LAA morphological classification was exceptionally high, with an overall agreement rate of 99% (459 out of 463 cases). The strength of agreement was quantified by a Cohen’s kappa coefficient of 0.982 (*p* < 0.001), indicating near-perfect reliability across the four morphological subtypes. Baseline clinical characteristics were largely similar across the four groups (Table 1). There were no significant differences in age, sex distribution, or major comorbidities such as hypertension, diabetes, or prior stroke among the LAA morphology types (all *p* > 0.05). The proportion of patients with paroxysmal vs. persistent AF was also comparable (overall 67% paroxysmal; *p* = 0.186 across groups). One notable exception was a higher prevalence of chronic heart failure in the cactus group (27% of cactus patients vs. 10–13% in the other groups, *p* = 0.022). In addition, use of AAD before ablation differed by LAA type: the cactus group had the highest rate of pre-ablation antiarrhythmic therapy (85.5% of patients) while the windsock group had the lowest (51.7%), a significant variation (*p* = 0.006). Post-ablation antiarrhythmic drug usage during the blanking period was similar among the four groups (*p* = 0.332).

Baseline cardiac CT measurements of LAA anatomy showed some differences in morphology. The cactus-type LAA had the widest mean ostial diameter (21.3 ± 5.6 mm), which was significantly larger than that of the chicken-wing LAA (18.6 ± 4.9 mm; *p* = 0.001 for overall group comparison). The windsock-type LAA tended to have the longest appendage length (37.4 ± 7.3 mm, compared to 29.4 ± 7.8 mm in the shortest, cauliflower-type; overall *p* = 0.028). LAA morphology was also associated with differing complexity of lobes: windsock appendages most often had a single dominant lobe with no additional lobes (72% of windsock patients had 0 secondary lobes, versus 27–44% in the other groups; *p* < 0.001 for lobe count distribution). Despite these anatomical differences, the mean LAA volumes were comparable among the four morphologies (windsock 13.8 ± 6.6 mL vs. range of 11.9–13.7 mL in others; *p* = 0.299). The average distance from the LAA ostium to the LSPV was around 24–25 mm in all groups and did not differ significantly (*p* = 0.257). The cactus group did exhibit a larger mean left atrial volume on echocardiography/CT (151 ± 56 mL) compared to the chicken-wing group (128 ± 44 mL), with an overall group difference (*p* = 0.006), but this volumetric difference did not clearly translate into differing baseline AF type proportions or immediate procedure outcomes.

### 3.2. Recurrent Rate Among the Four Groups

**Figure 2:**
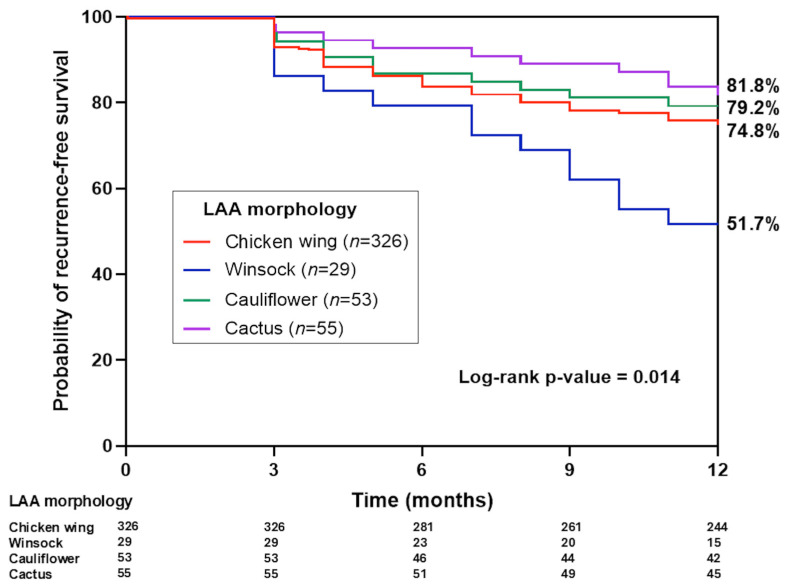
Kaplan–Meier curves for one-year atrial tachyarrhythmia recurrence by left atrial appendage (LAA) morphology. Patients with windsock-type LAA morphology exhibited a significantly higher cumulative incidence of atrial tachyarrhythmia recurrence at one year compared to the other morphologies (log-rank *p* = 0.014).

All patients completed at least 12 months of follow-up. Atrial tachyarrhythmia recurrence between 3 and 12 months after the index ablation was observed in 117 of the 463 patients, yielding an overall recurrence rate of 25.3%. The incidence of recurrence varied significantly according to LAA morphology group (*p* = 0.017). Patients with a windsock-type LAA experienced the highest 1-year recurrence rate at 48.3% (14 of 29 patients). In contrast, the chicken-wing group had a 25.2% recurrence rate (82 of 326), the cauliflower group was 20.8% (11 of 53), and the cactus group was 18.2% (10 of 55). Kaplan–Meier analysis of arrhythmia-free survival across the four morphologies (Figure 2) demonstrated that the windsock group had significantly shorter AF-free survival compared to the other LAA types (log-rank *p* = 0.014). In pairwise comparisons, windsock morphology was associated with a markedly higher likelihood of recurrence, whereas the other three LAA types showed relatively similar recurrence rates to each other over the 1-year follow-up.

### 3.3. Univariable Analysis and Multiple Logistic Regression Analysis for Arrhythmia-Free in 12 Months

Table 2 compares the basic characteristics of patients with and without one-year recurrence of atrial tachyarrhythmia following radiofrequency ablation. Among 463 patients studied, 117 experienced recurrence within one year. There were no significant differences in age, sex distribution, or major comorbidities—including hypertension, diabetes mellitus, chronic heart failure, and prior stroke—between patients with and without recurrence (all *p* > 0.05). However, patients with recurrence were significantly more likely to have persistent AF than those without recurrence (45.3% vs. 28.3%, *p* = 0.001). Additionally, post-ablation antiarrhythmic drug use was significantly more frequent in the recurrence group (81.2% vs. 60.7%, *p* < 0.001). Regarding anatomical parameters, significant differences were observed between groups in terms of LAA morphology distribution (*p* = 0.017), larger LAA origin size (20.0 ± 5.1 mm vs. 18.7 ± 5.3 mm, *p* = 0.011), greater LAA-to-LSPV distance (24.9 ± 4.6 mm vs. 23.9 ± 4.7 mm, *p* = 0.029), increased LAA volume (14.5 ± 6.4 mL vs. 13.1 ± 6.7 mL, *p* = 0.024), and increased left atrial volume (143.3 ± 51.9 mL vs. 128.9 ± 49.7 mL, *p* = 0.004) Univariable logistic regression was performed to identify predictors of AF recurrence at 3–12 months (Table 3). Traditional clinical risk factors, including age, sex, hypertension, diabetes, heart failure, and history of stroke, were not significantly associated with recurrence in unadjusted analyses. Baseline AF subtype exerted a significant influence on recurrence risk, with patients presenting with persistent AF exhibiting markedly higher odds of recurrence compared to those with paroxysmal AF (ORs = 2.096; 95% CIs: 1.360–3.230; *p* = 0.006). Additionally, the use of AADs during the blanking period emerged as a robust univariable predictor of recurrence. Patients requiring AAD therapy within this early post-ablation phase demonstrated a substantially increased likelihood of arrhythmia recurrence at one-year follow-up (ORs = 2.797; 95% CIs: 1.677–4.664; *p* < 0.001).

Several LAA-specific anatomical parameters also showed associations with recurrence on univariable analysis. A larger LAA orifice diameter was linked to higher recurrence risk (ORs = 1.047 per 1 mm increase in diameter; 95% CIs: 1.006–1.090; *p* = 0.023). The number of LAA lobes was relevant: in particular, the subgroup of patients with a bi-lobed LAA (two lobes) had higher odds of recurrence than those with a single-lobed appendage (ORs = 1.827; 95% CIs: 1.032–3.233; *p* = 0.038). Greater overall left atrial size was modestly associated with recurrence as well (ORs = 1.005 per mL increase in LA volume; 95% CIs: 1.001–1.009; *p* = 0.009). Notably, LAA morphology emerged as an important factor—having a windsock-type LAA was associated with significantly increased odds of recurrence compared to non-windsock morphologies (ORs = 2.722; 95% CIs: 1.227–6.040; *p* = 0.003). In contrast, other anatomical measures such as LAA total length, LAA-to-LSPV distance, and absolute LAA volume did not show significant univariable relationships with recurrence (the trend toward higher recurrence with larger LAA volume did not reach statistical significance). In addition, the combined non-chicken wing group did not significantly differ from the chicken wing group in predicting AF recurrence (ORs = 1.332; 95% CIs: 0.892–1.989; *p* = 0.161). We evaluated potential multicollinearity among LA volume, LAA volume, and AF type using VIFs. All VIF values were below 2.0, indicating no significant collinearity among these variables.

On multivariable logistic regression analysis including relevant covariates, three factors remained independent predictors of recurrence (Nagelkerke R^2^ value = 0.270). Persistent AF at baseline continued to confer a higher risk of recurrence at 1 year (ORs = 1.748; 95% CIs: 1.075–2.842; *p* = 0.024, persistent vs. paroxysmal). The requirement of antiarrhythmic drug use after ablation (during the initial 3-month blanking period) was independently associated with recurrence (ORs = 2.862; 95% CIs: 1.689–4.849; *p* < 0.001). Importantly, LAA morphology retained prognostic value in the adjusted model: windsock-type LAA remained a significant independent predictor of recurrence (ORs = 2.720; 95% CIs: 1.209–6.118; *p* = 0.016) when compared to the other LAA configurations. In the multivariable model, LAA orifice size was no longer a significant factor, suggesting its effect was mediated by or overlapped with other variables. Similarly, the association of having more than two LAA lobes with recurrence was attenuated and did not reach significance after adjustment. Left atrial volume showed an univariable link with outcomes but was not an independent predictor in the multivariable analysis. In addition to the logistic regression analysis, a multivariate Cox proportional hazards regression model was employed to further evaluate the independent predictors of AF recurrence. The results concurred with the findings from the logistic model, reaffirming the significance of the identified variables. Specifically, persistent AF was associated with an increased risk of recurrence (HRs = 1.450; 95% CIs: 1.069–1.967; *p* = 0.017). Post-ablation use of AADs emerged as a strong predictor (HRs = 2.186; 95% CIs: 1.547–3.089; *p* < 0.001), and the presence of windsock pulmonary vein morphology was likewise independently associated with recurrence risk (HRs = 1.874; 95% CIs: 1.153–3.045; *p* = 0.011). In summary, after controlling for potential confounders, the windsock LAA morphology, persistent AF type, and need for post-ablation antiarrhythmic drug therapy emerged as the key factors associated with atrial tachyarrhythmia recurrence following first-time catheter ablation.

## 4. Discussion

The most important finding of this study is that LAA morphology significantly predicts AF recurrence within one year after catheter ablation, with the windsock-type associated with the highest recurrence rate. After adjustment in multivariable analysis, windsock-type morphology remained an independent and significant predictor of recurrence. Additionally, persistent AF and the requirement for AAD during the blanking period were independently associated with increased recurrence risk. In contrast, other anatomical parameters of the LAA, such as orifice diameter, length, lobe number, and volume, although initially significant in univariable analysis, lost their predictive value upon multivariable adjustment. This association between appendage morphology and ablation outcome is biologically plausible given the unique electrophysiological features of the LAA. These findings suggest that specific LAA morphology—particularly windsock-type—may play a critical role in post-ablation AF recurrence, highlighting the potential value of morphology-guided ablation strategies to optimize treatment outcomes and minimize thromboembolic risk. Any such strategy would need to carefully consider thromboembolic risk, especially for high-risk LAA anatomies like the windsock.

Mechanistically, the association between LAA morphology and AF recurrence is plausible. LAA is a complex structure enriched with pectinate muscles, which can alter electrical conduction and contribute to the development of AT and AF [14,15]. In addition, the ligament of Marshall, which contains both sympathetic and parasympathetic nerve fibers, and the Bachmann bundle are located near the LAA [1,15]. Abnormal connections involving these structures may contribute to ectopic firing originating from the LAA [1]. In 2016, one trial involving 173 patients demonstrated that, in individuals with long-standing persistent AF, empirical electrical isolation of the LAA significantly improved the rate of freedom from AF during follow-up [2]. Thus, the LAA has emerged as a potential contributor to the pathophysiology and recurrence of AF.

The left atrial appendage (LAA) exhibits considerable anatomical variation, with four classic LAA morphologies that have been described—chicken-wing, windsock, cactus, and cauliflower—and these have been studied for their association with stroke risk [16]. After adjusting for CHA_2_DS_2_-VASc factors, chicken-wing anatomy was associated with significantly lower odds of stroke, whereas non-chicken-wing shapes (including windsock) conferred higher risk—in fact, windsock anatomy carried about a 4.5-fold higher odds of stroke/TIA history compared to chicken-wing in that study. A 2020 meta-analysis of 12 studies (3486 patients) confirmed this pattern: “non-chicken wing” LAA morphologies (windsock, cactus, cauliflower) had significantly more thromboembolic events than chicken wings [17]. Windsock-type LAA morphology empties less effectively, resulting in sluggish blood flow and stasis that increase thromboembolic risk. Transesophageal echocardiography studies revealed that non-chicken-wing LAA shapes (including windsock) had significantly lower flow velocities (<35 cm/s), with nearly a tenfold higher odds of low appendage emptying flow in these patients [16,17]. Additionally, the windsock anatomy typically has a wider orifice and a larger single-lobe volume, which further reduces flow ejection force and increases thromboembolic potential. Although these hemodynamic disadvantages have been studied mostly in the context of stroke risk, they may also reflect underlying structural remodeling or an electrophysiological substrate that predisposes to AF recurrence. This possibility aligns with our finding of higher AF recurrence in patients with a windsock LAA morphology.

Despite the clear differences in thromboembolic risk among LAA morphologies, translating this knowledge into routine clinical practice (such as empirically isolating the LAA during ablation) remains controversial. Studies examining adjunctive LAA isolation have shown inconsistent efficacy, and there are significant safety concerns with this approach. The BELIEF trial demonstrated improved arrhythmia-free survival at 2 years with adjunctive empirical LAA isolation in patients with longstanding persistent AF (76% vs. 56%) [2]. However, these findings have not been consistently replicated in subsequent studies. Romanov et al. reported no significant benefit from adding concomitant LAA occlusion to PVI (66% vs. 59% success rate at 24 months, *p* = 0.34) [18], and a 16-year single-center analysis observed true AF-triggering foci within the appendage in only 0.3% of cases [19]. Moreover, multiple reports indicate that LAA isolation can heighten thromboembolic risk unless rigorous stroke prophylaxis is maintained [3,20]. Additionally, a recent multicenter cryoballoon ablation study by Yorgun et al. reported a 7.2% incidence of thromboembolic events during long-term follow-up, predominantly in patients non-adherent to anticoagulation [21]. These safety concerns underline the necessity of rigorous stroke prophylaxis strategies, either via uninterrupted anticoagulation or LAA occlusion, whenever LAA isolation is performed.

Prior studies examining LAA anatomy and function as predictors of post-ablation AF recurrence have yielded mixed results. Gong et al. reported that the classic “chicken-wing” LAA morphology was associated with the highest recurrence rates (approximately 68% of patients) after catheter ablation [11]. In contrast, a study revealed that larger LAA volume independently increased AF recurrence risk by about 34% after multivariate adjustment [9]. Similarly, Pongratz et al. identified LAA volume—but not morphology—as an independent predictor of ablation failure [22]. Moreover, a recent meta-analysis by Papathanasiou et al. concluded that LAA morphology itself did not predict recurrence, emphasizing instead functional measures such as reduced LAA emptying velocity and ejection fraction as correlates of AF persistence [9]. These findings highlight the complexity of interpreting LAA characteristics, with discrepancies likely stemming from variations in patient cohorts, imaging techniques, and statistical methodologies. Surgical LAA occlusion (e.g., with clip devices) might represent a promising adjunctive therapy [23]. This strategy could potentially reduce thromboembolic risk and suppress non-PV ectopic triggers. Nevertheless, current evidence supporting this approach remains limited, and further large-scale studies are needed to validate efficacy and clearly define suitable patient populations.

In the current study, however, we identified LAA morphology—specifically the windsock-type—as significantly associated with AF recurrence within one year after catheter ablation. Poor LAA functions, including LAA ejection fraction or slow emptying flow, larger LAA volumes, and orifice areas, were all associated with higher AF recurrence rates [9]. Contrary to previous findings emphasizing chicken-wing morphology [11], our results indicate that the windsock-type confers the highest recurrence risk. This discrepancy may be attributed to differences in imaging modalities and patient number. Compared to two-dimensional transesophageal echocardiography, multidetector computed tomography provides a more comprehensive evaluation of LAA morphology and even intracardiac structures [13]. The divergence between CT- and TEE-derived LAA morphological classifications likely reflects differences in imaging resolution and viewing geometry. Multidetector CT provides true three-dimensional volumetric data that allow full visualization of the LAA, including its distal lobes, orientation, and complex internal structure. In contrast, TEE is limited by acoustic window dependency and two-dimensional slice acquisition, which may obscure secondary lobes or distort the perceived appendage shape depending on probe angulation. Consequently, CT-based morphology assessment can reveal a more detailed and reproducible representation of anatomical heterogeneity, potentially explaining the discrepancy between our CT-based findings and prior TEE-based studies. Our multivariable analysis further confirmed that other anatomical parameters, such as LAA length, volume, orifice size, and lobe number, were not independently predictive after adjustment, suggesting that specific LAA morphology alone may reflect intrinsic arrhythmogenic properties, possibly related to substrate heterogeneity or latent trigger activities within the appendage. Alternatively, it is also possible that the windsock configuration may not be a direct causal factor for AF recurrence but rather a morphological manifestation of advanced atrial remodeling. The elongated and dilated shape of the windsock LAA could reflect cumulative atrial structural changes, such as chamber dilation and wall thinning, that accompany long-standing AF. Therefore, the observed association between windsock morphology and recurrence risk may partly represent the underlying remodeling severity rather than a purely anatomical cause. Longitudinal imaging studies assessing temporal changes in LAA morphology during AF progression would help clarify this causal direction.

In addition to LAA morphology, other clinical factors including persistent AF and the need for AAD therapy in the blanking period emerged as important predictors of recurrence in our study. Moreover, structural remodeling, including atrial dilation and fibrosis, plays a crucial role in AF pathogenesis. Compared to sinus rhythm, AF is associated with chamber dilation and reduced pectinate muscles [13]. Beyond contributing to the onset of arrhythmia, persistent AF also leads to significant intercellular space expansion and interstitial fibrosis, increasing the likelihood of AF recurrence after catheter ablation [24]. These findings underscore the multifactorial nature of AF recurrence and support a tailored ablation approach. In this context, our findings suggest that a selective, rather than routine, approach to LAA isolation could be warranted, particularly for patients with a windsock-type morphology, who appear to have the highest risk for AF recurrence.

Although follow-up included scheduled visits and symptom-triggered evaluations, recurrence ascertainment was partly dependent on the presence of symptoms. This approach may have biased recurrence detection toward symptomatic cases, thereby underestimating the true incidence of AF recurrence, particularly for subclinical or silent AF episodes. Future studies employing continuous or implantable rhythm monitoring could provide a more accurate estimation of recurrence burden.

Our findings support a personalized approach to AF ablation, guided by detailed evaluation of LAA morphology. Specifically, the windsock-type LAA emerged as a notable anatomical risk factor for post-ablation arrhythmia recurrence. This underscores a potential paradigm shift from uniform treatment protocols toward anatomy-informed strategies that incorporate morphological phenotyping into procedural planning. By moving beyond traditional metrics such as LAA size or electrical isolation, our study introduces structural configuration as a clinically relevant marker. These insights warrant prospective validation to determine whether morphology-guided ablation or appendage management can enhance rhythm control and reduce thromboembolic risk in AF populations.

## 5. Limitations

This was a single-center study conducted in an Asian population, which may limit the generalizability of the findings to other populations. Since this was a retrospective study, there is an inherent risk of unmeasured confounding factors. In addition, the windsock-type LAA group was relatively small—potentially reducing statistical power and increasing the risk of a biased or imprecise estimate of recurrence in that subgroup. This subgroup imbalance (*n* = 29 vs. 326 in the chicken-wing group) may also lead to overestimation of effect size and should be interpreted with caution. Finally, follow-up was largely symptom-driven rather than through continuous monitoring (only approximately 40% of patients underwent scheduled Holter monitoring at predefined intervals). As a result, the true recurrence incidence might be underestimated, particularly given that a significant proportion of AF episodes can occur without symptoms (silent AF). These limitations underscore the need to carefully interpret our results and warrant confirmation in larger, prospective multicenter studies. Furthermore, 48 patients were lost to follow-up (chicken-wing 35 [72.9%], windsock 2 [4.2%], cauliflower 4 [8.3%], cactus 7 [14.6%]). The distribution was similar to that of the analyzed cohort, suggesting that the loss group was not systematically biased. Thus, while this limitation may introduce a minor risk of Neyman bias, the potential impact on the main findings is likely minimal. Moreover, as a retrospective study spanning over a decade, residual confounding related to operator experience, procedural techniques, and changes in antiarrhythmic prescribing practices cannot be fully excluded. Evolution in ablation technologies and clinical decision making over time may have subtly influenced recurrence outcomes despite statistical adjustment.

## 6. Conclusions

In this retrospective single-center study, we demonstrated that LAA morphology, particularly the windsock-type, was associated with a higher risk of AF recurrence within one year after first-time catheter ablation. Further prospective studies are needed to validate these findings and to explore whether LAA-directed strategies could improve clinical outcomes in this patient population.

## Figures and Tables

**Table 1:** Basic characteristics of the patients with different left atrial appendage morphology.

Variables	Chicken Wing (*n* = 326)	Windsock (*n* = 29)	Cauliflower (*n* = 53)	Cactus (*n* = 55)	*p*-Value
Age (years)	59.9 ± 10.8	61.8 ± 11.8	62.5 ± 10.5	61.4 ± 9.6	0.316
Men	205 (62.9)	16 (55.2)	33 (62.3)	34 (61.8)	0.878
Hypertension	178 (54.6)	17 (58.6)	28 (52.8)	27 (49.1)	0.835
Diabetes mellitus	70 (21.5)	3 (10.3)	11 (20.8)	15 (27.3)	0.355
CHF	39 (12.0) ^a^	3 (10.3) ^ab^	7 (13.2) ^ab^	15 (27.3) ^b^	0.022
Stroke	32 (9.8)	6 (20.7)	4 (7.5)	6 (10.9)	0.272
AF type					0.186
Paroxysmal	228 (69.9)	15 (51.7)	34 (64.2)	35 (63.6)	
Persistent	98 (30.1)	14 (48.3)	19 (35.8)	20 (36.4)	
AADs use					
Pre-ablation	236 (72.4) ^ab^	15 (51.7) ^a^	34 (64.2) ^ab^	47 (85.5) ^b^	0.006
Post-ablation	208 (63.8)	19 (65.5)	36 (67.9)	42 (76.4)	0.332
LAA structure					
LAA orifice size (mm)	18.6 ± 4.9 ^a^	20.8 ±5.0 ^ab^	18.8 ±6.5 ^ab^	21.3 ± 5.6 ^b^	0.001
LAA length (mm)	35.6 ± 17.7 ^a^	37.4 ± 7.3 ^ab^	29.4 ± 7.8 ^b^	32.6 ± 8.2 ^ab^	0.028
LAA lobe number					<0.001
0 lobe	89 (27.3)	21 (72.4)	16 (30.2)	24 (43.6)	
1 lobe	70 (21.5)	1 (3.4)	8 (15.1)	20 (36.4)	
2 lobes	121 (37.1)	6 (20.7)	17 (32.1)	8 (14.5)	
≥3 lobes	46 (14.1)	1 (3.4)	12 (22.6)	3 (5.5)	
LAA to LSPV distance (mm)	23.9 ± 4.4	25.4 ± 5.2	24.3 ± 5.9	24.8 ± 4.6	0.257
LAA volume (mL)	13.7 ± 6.6	13.8 ± 6.6	11.9 ± 7.0	13.2 ± 6.6	0.299
LA volume (mL)	127.7 ± 44.2 ^a^	133.6 ± 53.8 ^ab^	142.7 ± 71.1 ^ab^	151.0 ± 56.4 ^b^	0.006

The data are presented as means ± standard deviations or numbers (percentages). Different letters (^a^ and ^b^) in the columns denote statistically significant differences (*p* < 0.05, Bonferroni). AADs = antiarrhythmic drugs; AF = atrial fibrillation; CHF = congestive heart failure; LAA = left atrial appendage; LSPV = left superior pulmonary vein.

**Table 2:** Basic characteristics of the studied patients with and without one-year recurrence of atrial tachyarrhythmia.

Variables	Recurrence (*n* = 117)	No Recurrence (*n* = 346)	*p*-Value
Age (years)	61.4 ± 10.2	60.2 ± 10.9	0.305
Men	66 (56.4)	222 (64.2)	0.135
Hypertension	62 (53)	188 (54.3)	0.801
Diabetes mellitus	26 (22.2)	73 (21.1)	0.798
Congestive heart failure	16 (13.7)	48 (13.9)	1.000
Stroke	8 (6.8)	40 (11.6)	0.164
AF type			0.001
Paroxysmal	64 (54.7)	248 (71.7)	
Persistent	53 (45.3)	98 (28.3)	
Antiarrhythmic drug use			
Pre-ablation	80 (68.4)	252 (72.8)	0.355
Post-ablation	95 (81.2)	210 (60.7)	<0.001
LAA structure			
LAA morphology			0.017
Chicken wing	82 (70.1)	244 (70.5)	
Windsock	14 (12.0)	15 (4.3)	
Cauliflower	11 (9.4)	42 (12.1)	
Cactus	10 (8.5)	45 (13.0)	
LAA orifice size (mm)	20.0 ± 5.1	18.7 ±5.3	0.011
LAA length (mm)	34.9 ± 6.7	34.5 ± 17.6	0.417
LAA lobe number			0.197
0 lobe	39 (33.3)	111 (32.1)	
1 lobe	17 (14.5)	82 (23.7)	
2 lobes	43 (36.8)	109 (31.5)	
≥3 lobes	18 (15.4)	44 (12.7)	
LAA to LSPV distance (mm)	24.9 ± 4.6	23.9 ± 4.7	0.029
LAA volume (mL)	14.5 ± 6.4	13.1 ± 6.7	0.024
LA volume (mL)	143.3 ± 51.9	128.9 ± 49.7	0.004

The data are presented as means ± standard deviations or numbers (percentages). AF = atrial fibrillation; LAA = left atrial appendage; LSPV = left superior pulmonary vein.

**Table 3:** Univariable and multivariable analysis for predictors of one-year atrial tachyarrhythmia recurrence.

Variables	Univariable		Multivariable	
	ORs (95% CIs)	*p* Value	ORs (95% CIs)	*p* Value
Age	1.010 (0.991–1.031)	0.304		
Men	0.723 (0.472–1.107)	0.136		
Hypertension	0.947 (0.622–1.442)	0.801		
Diabetes mellitus	1.068 (0.644–1.773)	0.798		
CHF	0.983 (0.535–1.808)	0.957		
Stroke	0.561 (0.255–1.237)	0.152		
AF type				
Persistent (vs. paroxysmal)	2.096 (1.360–3.230)	0.006	1.748 (1.075–2.842)	0.024
AADs use				
Pre-ablation	0.807 (0.511–1.273)	0.355		
Post-ablation	2.797 (1.677–4.664)	<0.001	2.862 (1.689–4.849)	<0.001
LAA structure				
LAA orifice size	1.047 (1.006–1.090)	0.023	1.010 (0.950–1.073)	0.756
LAA length	1.001 (0.989–1.014)	0.835		
LAA lobe number (≥2 vs. 1)	1.827(1.032–3.233)	0.038	1.822(0.998–3.326)	0.051
LAA to LSPV distance (mm)	1.043 (0.998–1.090)	0.061	0.982 (0.925–1.042)	0.546
LAA volume (mL)	1.031 (1.000–1.062)	0.051	0.997 (0.953–1.043)	0.997
LAA morphology				
Chicken wing	1	-		
Windsock	2.722 (1.227–6.040)	0.014		
Cauliflower	1.017 (0.564–1.833)	0.956		
Cactus	1.194 (0.672–2.121)	0.545		
Chicken wing (vs. non-chicken wing)	1.332 (0.892–1.989)	0.161		
Windsock (vs. non-windsock)	2.722 (1.227–6.040)	0.003	2.720 (1.209–6.118)	0.016
LA volume (mL)	1.005 (1.001–1.009)	0.009	1.004 (0.998–1.010)	0.154

Univariable and multivariable logistic regression were conducted to assess associations between recurrence of atrial tachyarrhythmia and clinical factors, including baseline characteristics, AF type, and left atrial and LAA parameters. Results are presented as odds ratios (ORs) with 95% confidence intervals (CIs) and corresponding *p* values. AADs = antiarrhythmic Drugs; AF = atrial fibrillation; CHF = congestive heart failure; LAA = left atrial appendage; LSPV = left superior pulmonary vein.

## Data Availability

The original contributions presented in this study are included in the article. Further inquiries can be directed to the corresponding authors.

## Abbreviations

PV, pulmonary vein; LAA, left atrial appendage; AF, atrial fibrillation; CT, computed tomography; LSPV, left superior PV; PVI, pulmonary vein isolation; AT, atrial tachycardia; AFL, atrial flutter; ECG, electrocardiogram; AAD, antiarrhythmic drugs; OR, odds ratio; CI, confidence interval.
